# Supplementary material for: Free Bilirubin Induces Neuro-Inflammation in an Induced Pluripotent Stem Cell-Derived Cortical Organoid Model of Crigler-Najjar Syndrome
Source: Cells. 2023 Sep 14;12(18):2277. doi: 10.3390/cells12182277 (PMC10527749; doi:10.3390/cells12182277)
Supplement: Supplementary file 1 [file cells-12-02277-s001.zip › Revised Supplementary Figure Legend.pdf]

Supplementary Figure 1: Generation of UM51 and i705-C2 iPSC-derived HLCs to check the presence or expression of UGT1A1.

(a) (i.ii), **(b).** (i,ii) IF showed characterization of the iPSC-derived HLCs by using typical HLC markers. Both cell lines showed HNF4a, E-cadherin, CYP2D6, AFP and albumin protein expression. The UGT1A1 was expressed on the healthy line, but absent in the patient line. Scale bars depict 50 µm. **(c)** CYP3A4 and CYP2D6 assays were performed to check the functionality of the generated HLCs. **(d)** qPCR analysis showed reduced but presence of *UGT1A1* expression in mRNA level along with expression of other HLC markers, such as *AFP, albumin* and *CYP2D6*. Depicted values are mean of three different independent (n=3) experiments. Error bars depict ± 95% confidence interval. Values were normalized to *RPL0* (housekeeping gene) and subsequently to DMSO treated control organoids. **(e)** (i,ii) A very fade expression of UGT1A1 was observed in WB analysis. HEPG2 was used as a positive control. The bar graph represents the UGT1A1 expression in fold change. Values were normalized to GAPDH (housekeeping protein).

Supplementary Figure 2: The secretome analysis of each individual sample using Human XL Cytokine Array.

(a) Individual image of BF treated and non-treated Human XL cytokine array membranes for both cell lines and time points. **(b)** The complete heatmap of each condition, representing the secretome profile, which was obtained after analyzing the membrane dots. **(c)** mRNA expression of *VEGFA* (i) and *SHBG* (ii) after BF treatment. Depicted values are mean of three different independent (n=3) experiments. Error bars depict ± 95% confidence interval. Values were normalized to *RPL0* (housekeeping gene) and subsequently to DMSO treated control organoids.

Supplementary Figure 3: Selected GO terms with corresponding genes and KEGG pathways from the uniquely expressed genes post BF exposure.

(a) Selected GO terms showing the corresponding genes from the uniquely expressed genes after BF treatment on both cell lines at 72 hrs time point. **(b)** qPCR analysis showing an increase in mRNA expression of *NLRP3* (1.9-fold at i705-C2 24hrs),*CREB1* (1.15-fold at UM51 72hrs, i705-C2 24hrs) *, TNFSF12* (1.5-fold at i705-C2 72hrs), *AZI2* (1.2-fold at i705-C2 24hrs), and *MyD88* (1.2-fold at i705-C2 72hrs) for certain time points. These genes were selected from the mentioned GO terms. Depicted values are mean of three different independent (n=3) experiments. Error bars depict ± 95% confidence interval. Values were normalized to *RPL0* (housekeeping gene) and subsequently to DMSO treated control organoids. **(c)** Dot plot from KEGG associated pathways for healthy cell line, did not show an initiation of inflammatory related pathways at 24hrs.

Supplementary Figure 4: WB analysis and Dotplots of upregulated GO.BP terms from the common set of genes at 72hr post BF treatment.

(a). (b). (c): WB analysis showing CREB, Phospho-CREB and phospho-P38MAPK protein expression. **(a)** (i, ii) **(b)** (i, ii) The UM51 showed slight increase in protein expression for both CREB (1.3-fold) and phosphor-CREB (1.12-fold) at 24 hrs, while the i705-C2 line did not show an increase. **(c)** (i) 1.25-fold increase in phospho-P38MAPK expression was observed in the patient line-derived samples for both 24 and 72 hrs time point. **(a)** (iii), **(b)** (iii), **(c)** (ii), Bar graphs showing the protein expression in fold change. Values were normalized to ß-Actin **(a), (b)** and GAPDH **(c)** (housekeeping proteins) and subsequently to DMSO treated control organoids. **(d)** (i, ii) GO terms of the common set of genes showing the neurodevelopmental pathways to be upregulated for both cell lines at 72 hrs post BF treatment.

Supplementary Figure 5: Immunofluorescence staining showing the expression of γH2AX at 24hrs post BF exposure and heatmap showing necroptosis associated gene expression.

(a), (b) 24 hrs post treatment, in both cell lines IF showed a slight increase in the presence of DNA damage and repair related and apoptotic marker yH2AX positive cells compared to the DMSO treated control. **(a)** (i, ii) Scale bars depict 100 µm. **(b)** (i, ii) Bar graphs indicate quantification of yH2AX staining (N=6). Error bars depict mean ± SD. Asterisk depicts significance, which is determined by p-value ≤ 0.05. Significance in comparison to control was calculated by using Student`s unpaired two sample t- test.

(c) Pearson heatmap obtained from transcriptome analyses, presented necroptosis associated genes. Regardless of the BF treatment, the patient samples clustered separately in the heatmap than the healthy line derived samples. The upregulated genes for the patient line are associated with inflammation, while the upregulated genes for the healthy line are associated with apoptosis.
