# Supplementary figures and images for: Free Bilirubin Induces Neuro-Inflammation in an Induced Pluripotent Stem Cell-Derived Cortical Organoid Model of Crigler-Najjar Syndrome
Source: Cells. 2023 Sep 14;12(18):2277. doi: 10.3390/cells12182277 (PMC10527749; doi:10.3390/cells12182277)

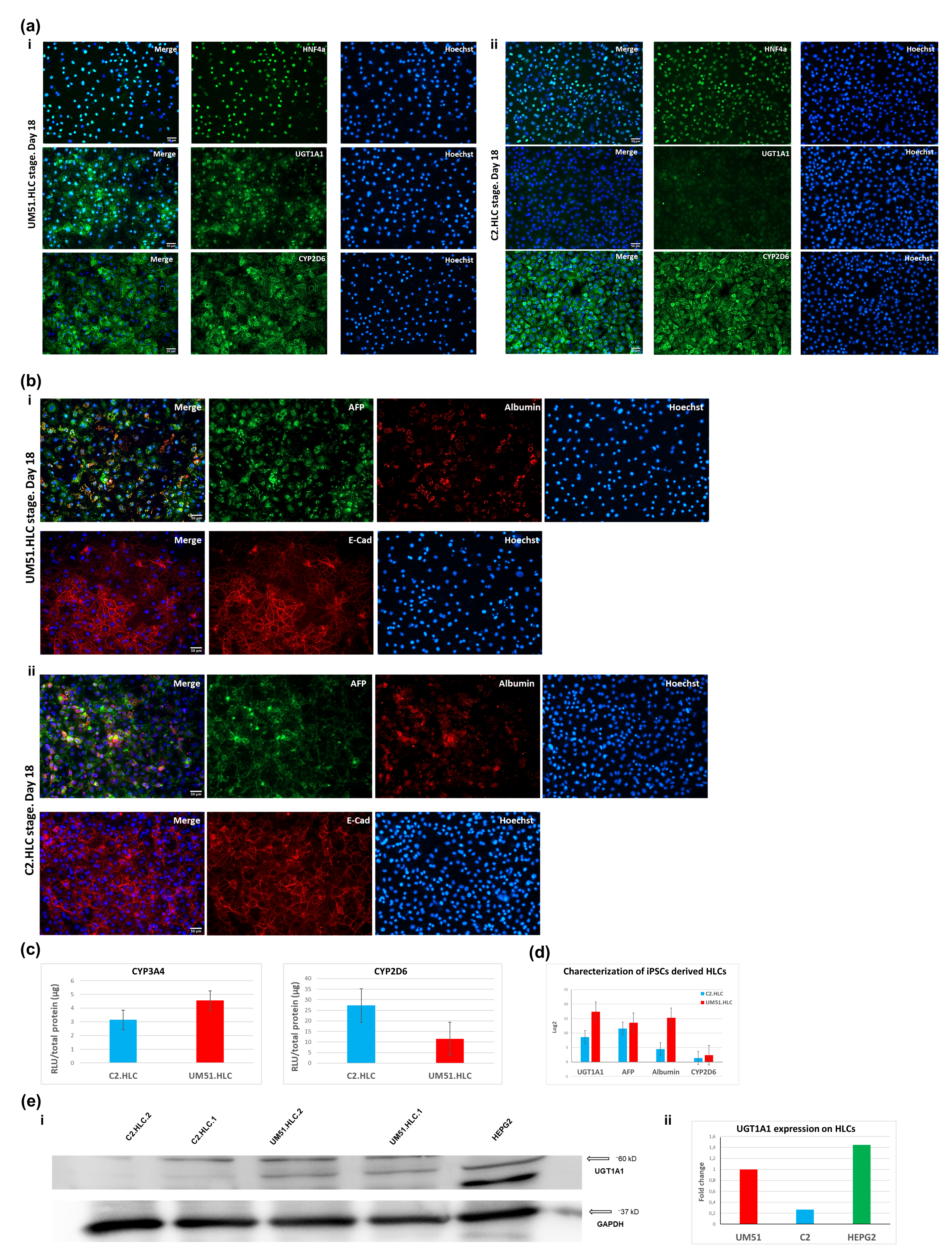

Supplement: Supplementary file 1 [file cells-12-02277-s001.zip › Supplementary Fig 1.png]

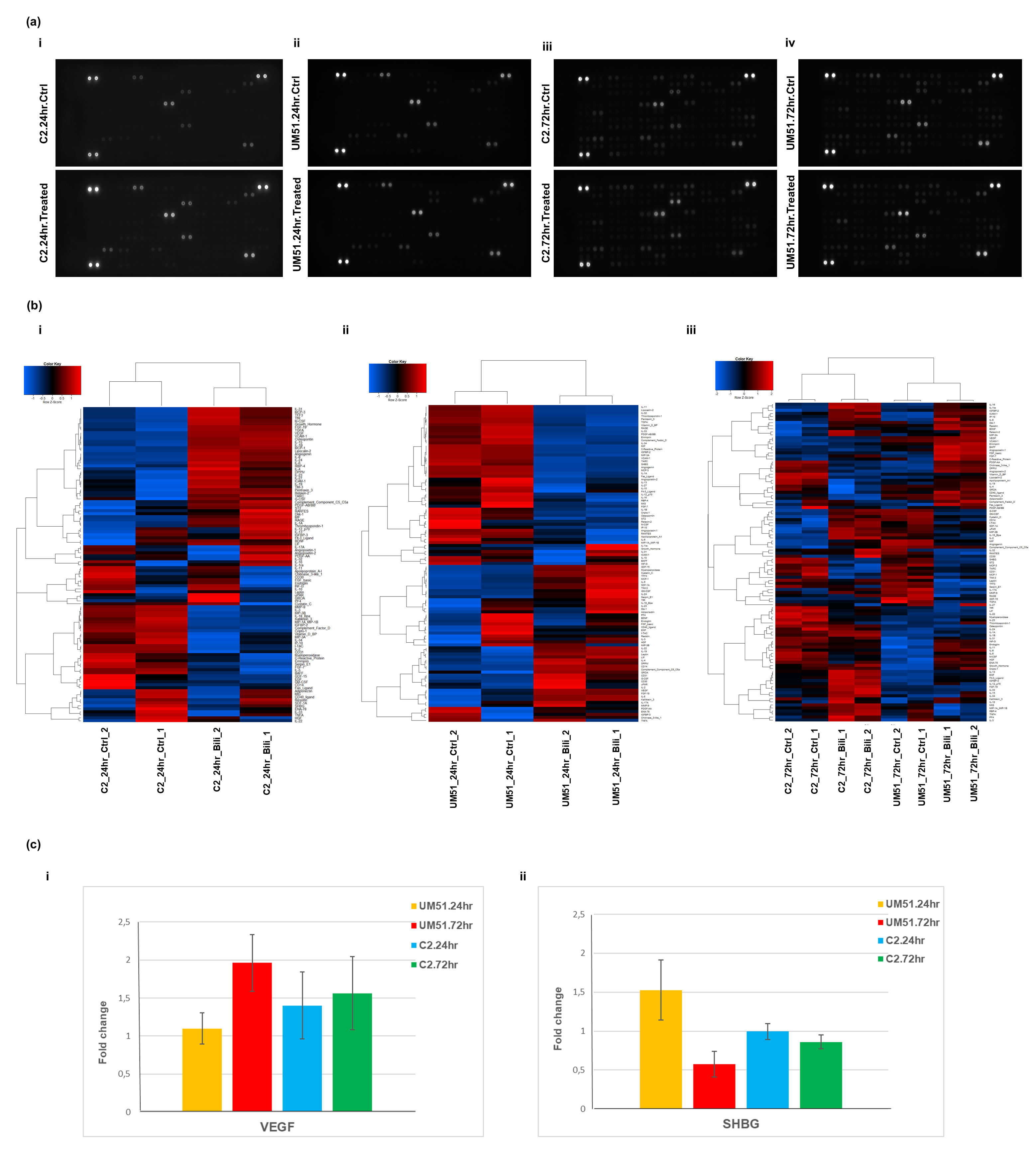

Supplement: Supplementary file 1 [file cells-12-02277-s001.zip › Supplementary Fig 2.jpg]

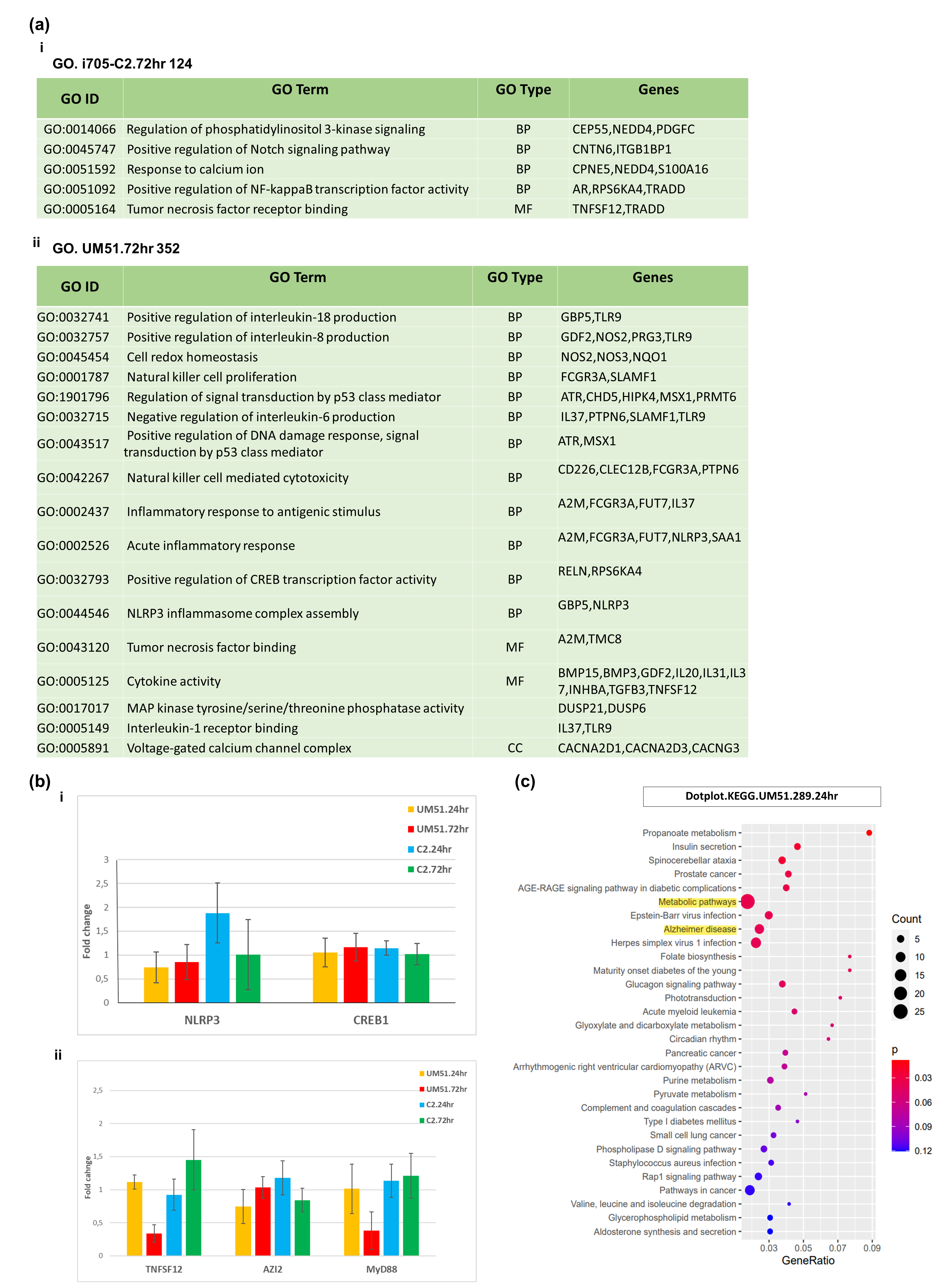

Supplement: Supplementary file 1 [file cells-12-02277-s001.zip › Supplementary Fig 3.png]

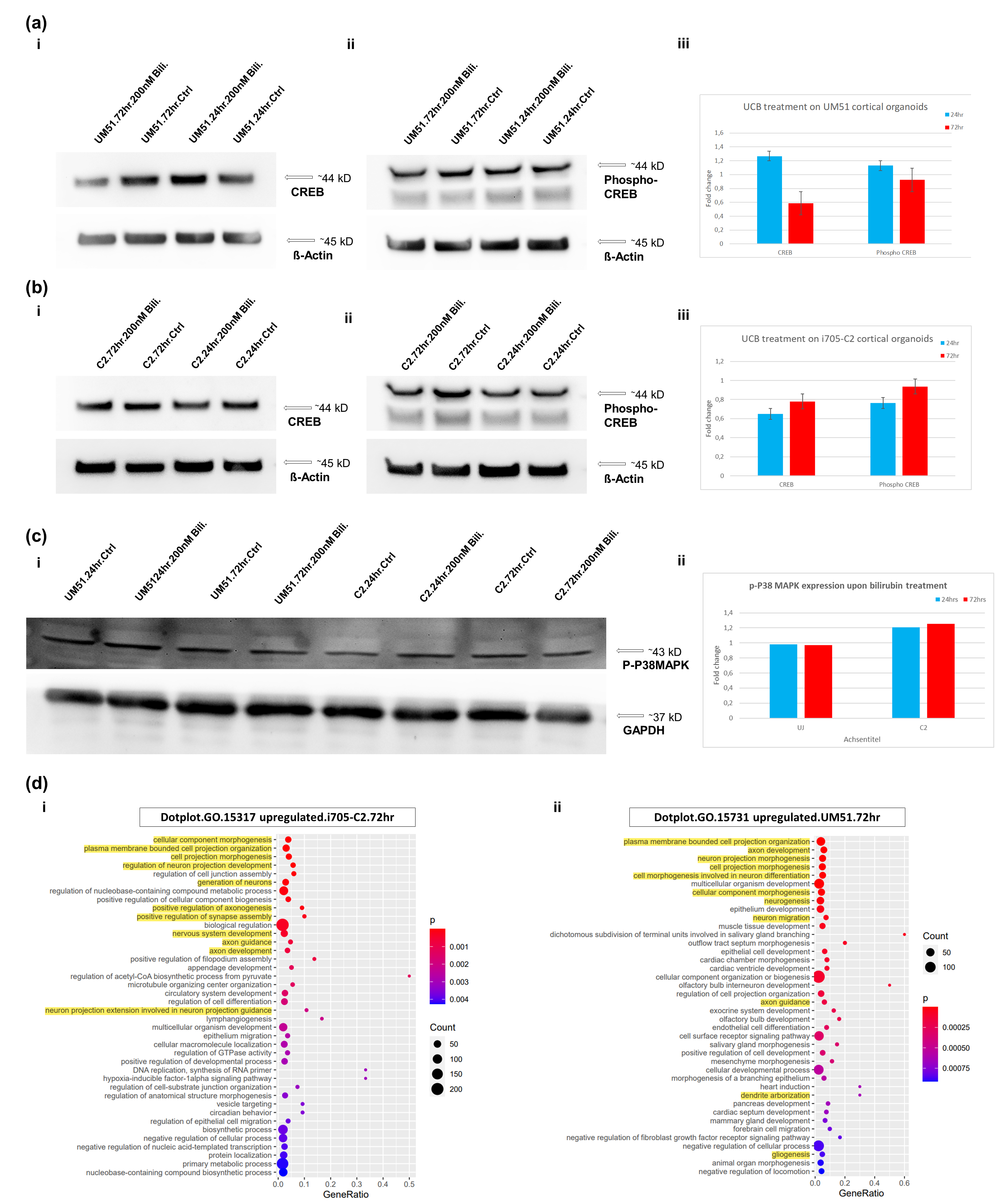

Supplement: Supplementary file 1 [file cells-12-02277-s001.zip › Supplementary Fig 4.png]

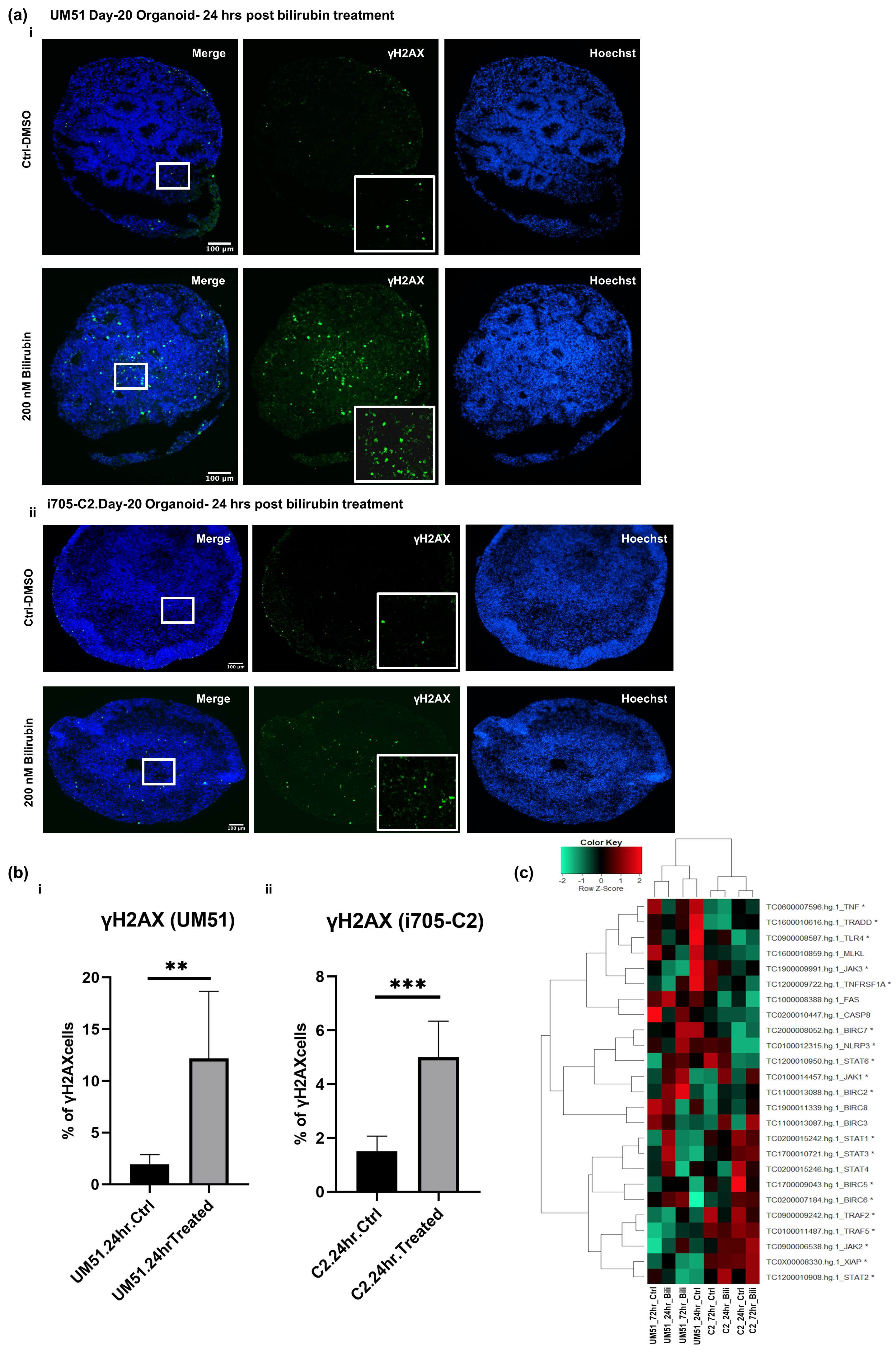

Supplement: Supplementary file 1 [file cells-12-02277-s001.zip › Supplementary Fig 5.png]
